# Peptic ulcer perforation after cesarean section; case series and literature review

**DOI:** 10.1186/s12893-020-00732-9

**Published:** 2020-05-24

**Authors:** Mahboobeh Shirazi, Mehnoosh Tork Zaban, Sriharsha Gummadi, Marjan Ghaemi

**Affiliations:** 1 Maternal, Fetal and Neonatal Research Center, Tehran University of Medical Sciences, Tehran, Iran; 2 Breast feeding Research Center, Tehran University of Medical Sciences, Tehran, Iran; 3 Department of Radiology, Thomas Jefferson University, Philadelphia, PA USA; 4 Department of Surgery, Lankenau Medical Centre, Wynnewood, PA USA; 5 Kamali Hospital, Alborz University of Medical Sciences, Karaj, Tehran Iran

**Keywords:** Perforated peptic ulcer, Cesarean section, Maternal mortality

## Abstract

**Background:**

Peptic ulcer perforation in the early post-cesarean period is rare but may result in maternal mortality.

**Case presentation:**

Four cases of post-cesarean peptic ulcer perforation are presented. In all four patients, presentations include peritoneal signs such as acute abdominal pain and progressive distention, hemodynamic instability and intraperitoneal free fluid by ultrasound. Laparotomy and repair were done in all 4 cases. There were 2 maternal deaths. We also have reviewed English literature for the similar cases reported from 1940 to March 2019.

**Conclusion:**

New onset tachycardia, abdominal pain and progressive distension after cesarean section without congruent changes in hemoglobin should raise concerns for intra-abdominal emergencies including perforated peptic ulcer. Early use of ultrasound should be considered to assist in diagnosis. Coordinated care by an obstetrician and a general surgeon is necessary in presence of any unusual postoperative abdominal pain. Early recognition of the disease is imperative to limit the surgical delay and to improve the outcomes.

## Background

Caesarean section is the most common obstetrical procedure worldwide. Post-cesarean section surgical emergencies are rare [1]. Re-operation after cesarean section is performed at 0.5–1.5% of cases, usually by abdominal laparotomy [2]. Post cesarean gastrointestinal complications are extremely rare and mostly involve the large bowel. Perforated peptic ulcer (PPU) following cesarean section is rare and information regarding this diagnosis is lacking [3]. In this case series, we provide four cases that underwent early post cesarean section re-laparotomy due to PPU and also, we reviewed English literature for the similar cases reported from 1940 to March 2019.

Data were extracted from local maternal mortality and morbidity committee in the city of Tehran from March 2015 to April 2018 among 608.000 deliveries. There was no vaginal delivery complicated by peptic ulcer meanwhile. The clinical manifestations, the diagnostic and therapeutic approaches, and the outcomes are detailed. We have also performed a PubMed, Ovid Medline, and google scholar literature search of English language articles from 1940 to March 2019 using keywords: “peptic ulcer perforation” “gastric ulcer” “duodenal ulcer” and “cesarean section” or” abdominal delivery”. Approval from our institution’s review board and local ethics committee was obtained. Written consent was signed upon admission by all patients included in this study to use their information in research studies.

## Case presentation

### First case

**Fig. 1:**
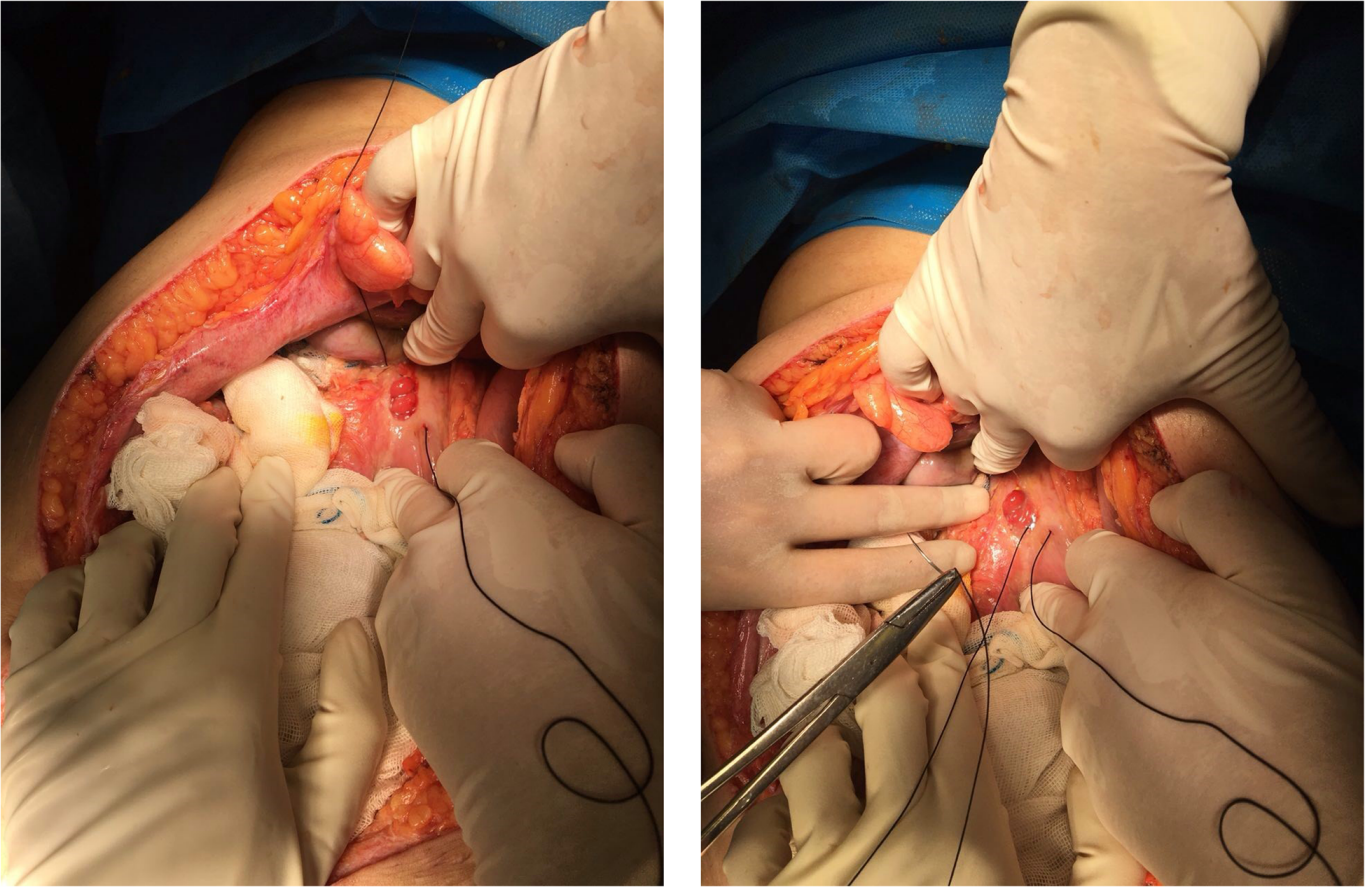
A 2*2 centimeter perforation in anterior stomach wall resulting in peritonitis. The perforated area was being sutured

A 35 year old G2P1 (Gravida 2 Para 1) pregnant woman was admitted to a university hospital in 38 weeks of gestational age due to labor pain. An uneventful cesarean section was performed due to the previous cesarean section and there were no extensive adhesions. Due to patient’s request, she was discharged 24 h post operation after physician examination, in normal general condition and oral NSAID pain killers were prescribed. She was readmitted on the 3rd day postpartum for the left upper quadrant abdominal pain, abdominal distension, and tachycardia with pulse rate of 108 per minute. Additionally, she endorsed nausea, vomiting, and constipation. An emergent abdominal ultrasonography was performed which revealed multiple gas-filled bowel loops and large amount of free fluid in the abdominal cavity. Re-laparotomy via Pfannenstiel incision was performed after 7 h of admission and a 2 × 2 cm perforation in anterior stomach wall was demonstrated (Fig. 1). The perforated area was repaired by general surgeon. She had an uneventful postoperative recovery and was discharged 7 days later.

### Second case

A 30 year old G3P1(Gravida 3 Para1) pregnant woman had a scheduled cesarean section at term due to the previous cesarean section. In the morning of the second day postpartum, she experienced a sudden onset severe abdominal pain, chest pain and dyspnea. Her vital signs were recorded as 105/min for pulse rate, 110/70 mmHg for blood pressure and 18/min for the respiratory rate. O2 saturation was normal with the Hb level of 10.5 g/dl. The patient’s hemodynamics worsened (BP = 80/55, PR = 130/min) in the afternoon and abdominal pain and dyspnea was reduced. Bedside abdominal ultrasound was requested and revealed massive intra peritoneal fluid. Re-laparotomy after 3 h of admission via Pfannenstiel incision was performed by the obstetrician with the probable diagnosis of hemoperitoneum. A general surgeon was attended after detectinggastric fluid and he explored the abdomen via midline incision. Five liters of gastric fluid was collected in abdominal cavity along with a duodenal perforation that completely repaired by general. The patient was transferred to the intensive care unit (ICU) and ultimately discharged 1 week later in stable condition.

### Third case

A 34 year old G1 (gravida1) pregnant woman was admitted with complaints of a headache, vertigo, and vomiting in 36 weeks of gestational age. The blood pressure was 140/90 mmHg upon admission, and the urinalysis showed 2+ proteinuria. However, on hospital day 1, the patient was taken for the emergent cesarean section due to preterm labor pain and fetal distress. Her blood pressure rose to 150/95 mmHg postoperatively and the loading dose of magnesium sulfate was prophylactically administered. On postoperative night 2, she developed worsening abdominal pain and distension, obstipation and new onset hypotension (100/60 mmHg). An upright abdominal x-ray was performed and demonstrated air-fluid levels. A bedside ultrasound was performed and showed massive intraperitoneal fluid. Re-laparotomy was performed by obstetrician within 6 hours from the onset of pain, with the probable diagnosis of hemoperitoneum. General surgeon was consulted who explored the abdomen via midline incision and found a pre-pyloric ulcer and repaired the perforation. The patient was transferred to ICU but required mechanical ventilation due to decreased level of consciousness. Postoperatively, the patient developed high fever despite broad-spectrum antibiotics. Blood cultures were positive for *E. coli*. The patient subsequently developed acute respiratory distress syndrome complicated by pneumothoraces requiring bilateral tube thoracostomy. She died on post-operative day 8. Based on autopsy results, the cause of death was reported as disseminated abdominal infection.

### Fourth case

A 32 year old G2P1 (gravida 2 para1) pregnant woman was admitted for a scheduled cesarean section at term due to the previous cesarean section. An uneventful cesarean section was performed. In ten hours post-operation, patient developed sudden abdominal pain, distension and tachycardia. She had bowel function but worsening abdominal distension prompted further clinical evaluation. Within two hours, the patient became cyanotic and clinical presentations of the cold phase of septic shock appeared. A bedside ultrasound was performed, demonstrating a large amount of intra-peritoneal free fluid. Re-laparotomy was performed rapidly by the obstetrician via Pfannenstiel incision when gastric fluid and food particles were encountered within the abdomen. A general surgeon was consulted, and explored the abdomen via midline incision, found a perforated duodenal ulcer and repaired the perforation. The patient developed cardiac arrest during the operation and subsequently died.

## Discussion and conclusion

Perforated peptic ulcer (PPU) is a surgical emergency associated with short-term mortality in up to 30% of patients [4]. It accounts for one of the highest mortality rates after emergency surgeries overall [5]. In a cohort study of 2668 patients treated surgically for PPU, every hour of surgical delay was associated with a 2. 4% decreased probability of 30-day survival. Therefore, it is imperative to limit the surgical delay in any patient with suspected PPU [6]. PPU represents a rare but potentially mortal diagnosis after the cesarean section, particularly in the early postpartum period [7].

In one study 69.0% of patients diagnosed with PPU had no previous history of treatment for peptic ulcer disease and 87.5% had reported medication history of non-steroidal anti-inflammatory drug (NSAID) usage [8]. In this case series, first case had a history of gastrointestinal discomfort prior to pregnancy which could be due to a peptic ulcer disease. It is imperative to ask about patients’ previous medical and medication history during prenatal visits, prescribe antacids and/or H2 blockers in case of gastroesophageal reflux and request Helicobacter pylori test if indicated. However, in presence of clinical signs and symptoms, the lack of a past medical history should not delay the diagnosis.

There is a classic triad of acute onset abdominal pain, tachycardia, and abdominal rigidity which is the hallmark of PPU. Tachycardia occurs due to the compensatory reflex regarding to severe pain, systemic inflammatory response from chemical peritonitis, and fluid deficit either due to the poor intake, vomiting or pyrexia [9].

In postpartum setting, acute abdominal pain of PPU may be confused with usual post-operative discomfort and may be subsided in patients who receive post cesarean narcotic analgesics, and any tenderness may be confused with local pain at the incision site [1]. However, new onset tachycardia and constant or increasing abdominal pain with progressive distention should prompt attention in post cesarean phase, since it may be easily misdiagnosed with paralytic ileus, which is not uncommon postoperatively [10].

Here, we reported 4 patients who developed abdominal pain in the early postpartum period between 10 hours to 3 days postpartum. All of them had acute abdominal pain and progressive abdominal distension, which was misdiagnosed as paralytic ileus in two. All four patients experienced tachycardia without primary changes in hemoglobin or blood pressure to prompt concern for hemorrhage. Dyspnea prompted an erroneous diagnosis of the pulmonary embolism (PE) in second case. Chest pain and dyspnea has been also reported in a 54 year old man as an unusual presentation of the perforated peptic ulcer [11].

It is believed that demonstration of free air on a plain abdominal upright X-ray is highly indicative of a perforated viscus organ and there is no other imaging modality necessary to use [12], but pneumoperitoneum (PP) after abdominal surgery represents a diagnostic challenge between normal PP following recent laparotomy and abnormal PP secondary to postoperative complications, such as gastrointestinal perforation [13]. In the postoperative setting, the radiological demonstration of PP in itself should not play a critical role in the decision whether exploration is indicated [14]. Grassi et al. found ultrasound (US) useful in PPU as it could identify the indirect findings of the perforation, such as the decreased peristalsis and the presence of free fluid between intestinal loops [15]. With the high index of suspicion, computed tomography after swallowing oral water-soluble contrast could be a good diagnostic tool for detecting PPU. An abdominal CT scan has additional value in ruling out other differential diagnoses such as abdominal aortic aneurysm or acute pancreatitis [12]. In this case report bedside US was performed in all four cases, and massive intraperitoneal fluid was reported as a common finding. In presence of free fluid in the abdominal ultrasound scan, comparing pre and post operational quantities of Hb level is important to estimate any blood loss, and may help in differentiating between hemoperitoneum and ascites. In case of ascites, as happened in our four cases, Hb level increases due to hemoconcentration. US has the advantages of being performed at bedside, increased patient tolerability and convenience, cost-effectiveness and absence of radiation exposure.

While laboratory data are not diagnostic for PPU, they are helpful for ruling out differential diagnoses such as acute pancreatitis [12]. Acute pancreatitis is highly suspicious when an acute onset epigastric pain is accompanied by an elevated level of serum lipase or amylase equal or greater than three times the upper limit of normal [16].

There are strong evidences for an association of comorbidity and use of NSAIDs with mortality following PPU [17]. cesarean section could be accounted as a comorbidity, due to extensive perioperative hemodynamic changes and increased stress, hence alongside with regular postoperative NSAID prescription it may result in mortality in a patient with PPU. It is highly recommended to administer antacids and H2 blockers 30 min pre-operation and avoid long perioperative NPO period in all cesarean sections. Authors avoid NSAIDs in patients with history of gastrointestinal problems during pregnancy and consider acetaminophen and celecoxib as first line non-narcotic post-cesarean pain killers for them. Actually, we did not administer PPI post section, maybe because for low dose and short time NSAID prescription; but it may be advisable to prescribe PPI post section for moderate and high risk patients.

In case of post-cesarean acute abdominal pain, a high index of suspicion by the obstetrician coupled with coordinated care by a general surgeon is necessary. Early diagnosis and prompt resuscitation and antibiotic therapy improve the outcomes of patients diagnosed with peptic ulcers perforation [12].

In our search in English literature, we identified and reviewed 8 reported cases of peptic ulcer perforation after the cesarean section, summarized in Table 1. The most common clinical signs and symptom were progressive abdominal distension, abdominal pain and tenderness, tachycardia and fever. Two patients were diagnosed with preeclampsia [10, 19] and one with eclampsia [20]. Signs and symptoms of peptic ulcer in these cases were primarily attributed to preeclampsia features.

**Table 1:** Summerized information about 8 reported cases of peptic ulcer perforation after cesarean section in English literature

Author, year	Maternal age	GA* at delivery	Reason for cesarean section	Signs & symptoms	Diagnostic tool	Type of surgery	Outcomes	Possible predisposing factor or relevant data
Engemise [10] 2009	29	35w	Non-assuring fetal cardiotocogram in the setting of preeclampsia and obstetric cholestasis	Day 5 pp.**: serous oozing was noted from the incision site Days 6-8 pp.: diffuse abdominal pain and progressive distension, coffee ground vomiting, decreased hemoglobin level Day 8-10 pp.: Tachycardia, diffuse abdominal tenderness, absent bowel sound, leukocytosis in the lab data	Abdominal and chest x-ray	Laparotomy (primary closure and omental patch) Re-laparotomy due to bilious leak from incision site, abdominal distension, and ongoing sepsis	Discharged 7 weeks postpartum in full recovery	Antenatal corticosteroid Oral NSAID 3 times a day for analgesia after delivery
Sule [18] 2010	27	Term	Obstructed labor	Day 3 pp.: loose stools, pyrexia and abdominal pains Day 4–11 pp.: progressive abdominal distension, pyrexia, and bilious fluid vomiting	Abdomino-pelvic ultrasound scan	Laparotomy (primary closure and omental patch) Re-laparotomy for intra-abdominal abscess drainage	Discharged 15 weeks after cesarean section in full recovery	*H. pylori* assay was positive
Ranganna [19] 2013	22	32w	No response to induction in the setting of eclampsia in a twin pregnancy	Epigastric discomfort since 2 days before admission Day 1 pp.: massive abdominal distension (loop thickening, ascites, and pleural effusion in abdominal scan) Day 3 pp.: mental disorientation and fall in the blood pressure, derangement of arterial blood gas, renal function, and coagulation profile Day 4 pp.: fever and greenish discharge from incision site (possible viscus perforation)	Computed tomography followed by peritoneocentesis (negative result)	Laparotomy (primary closure and omental patch)	Death 4 days after laparotomy	Vomiting for 1 week before admission, Epigastric discomfort since 2 days before admission
Maruyama [20] 2016	33	34w + 5d	Acute fatty liver of pregnancy	Day 2 pp.: bilateral vulvar hematoma Day 11 pp.: surgical evacuation of hematoma Day 13 pp.: Intermittent epigastric pain postpartum, massive abdominal distension, leukocytosis in the lab data Day 15 pp.: somnolence	Abdominal X-ray	Laparotomy (primary closure)	Discharged at the day 46 pp	Stress due to two consecutive surgeries (cesarean and hematoma drainage)
Ntirushwa [21] 2016	18	NR	NR	Day 4 pp.: Progressive abdominal distension, fever, tachycardia, dyspnea Day 5 pp.: fever, but clinical improvement in terms of abdominal pain and tenderness Day 6 pp.: massive abdominal distension	Bed side abdominal ultrasound scan	Laparotomy (five ascaris worms were in the peritoneal cavity and stomach was perforated)	Death 4 hours after laparotomy due to septic shock	Previous unresponsive to medication epigastric pain Intestinal Ascariasis
Ntirushwa [21] 2016	34	NR	Non-assuring fetal cardiotocogram in the setting of preeclampsia	Day 1 pp.: Edema, tachycardia, dyspnea, tachypnea, abdominal distension, and tenderness (diagnosed with pulmonary edema and treated accordingly) Day 2–10 pp.: clinical improvement Day 11 pp.: hypothermia, tachycardia, tachypnea, pus aspiration in peritoneocentesis	Abdominal ultrasound scan and peritoneocentesis (at day 2 and 11 pp)	Laparotomy (primary closure in two layers) Re-laparotomy due to explore suspected leakage of the gastric repair site.	Death 2 days after re-laparotomy due to septic shock	History of epigastric pain prior to cesarean delivery
Yildirim [18] 2016	29	Term	Cord prolapse	Day 2 pp.: abrupt generalized abdominal pain and distension, poor performance status, fever, tachycardia, bile-stained purulent fluid in peritoneocentesis	Peritoneocentesis Abdomino-pelvic ultrasonography Abdomino- thoracal computed tomography Tumor markers	Laparotomy (primary closure and biopsy of the gastric site) Definitive surgery 2 weeks after emergent laparotomy; radical distal gastrectomy, lymphadenectomy and gastro-jejunostomy, since the patient was diagnosed with gastric adenocarcinoma	Death 6 month after the initial diagnosis	History of epigastric pain, postprandial vomiting and weight loss over the last 3 months of pregnancy
Levin [3] 2018	22	34w	Breech presentation	Day 4 pp.: Abrupt upper abdominal pain and coffee ground vomiting, epigastric tenderness	Computed tomography Diagnostic laparoscopy	Laparotomy	Discharged 1 week after laparoscopy in full recovery	

*GA gestational age, **PP Post-Partum

Peritoneocentesis was performed in three cases [19–21] and was diagnostic in two [19, 21], resulting in laparotomy. Peritoneocentesis may be a practical tool to rule out hemoperitoneum. Finding intra-abdominal free fluid by ultrasound and ruling out the presence of blood can help the clinician to monitor the abdominal pain cases more cautiously.

Computed tomography was performed following an abrupt upper abdominal pain, coffee ground vomiting, and epigastric tenderness in one case, which revealed massive PP [3]. After a PPU was confirmed by laparoscopy, curative laparotomy was promptly done, and the patient survived without severe morbidity [3].

Across the review, 3 patients died in the first week after laparotomy [19, 20] and 1 died 6 months later due to gastric adenocarcinoma complications [21]. Additionally, 3 patients had prolonged hospitalization courses due to the secondary morbidity of PPU [10, 18, 22, 23]. Four patients required repeat exploration after initial laparotomy for PPU; 3 for abscess washout and drainage [10, 18, 19] and one for gastric adenocarcinoma staging [21]. The possibility of significant morbidity and mortality shows a need for high index suspicion by the obstetricians. In our study, the reason of dead in case 3 might be due to the leakage from previous perforation lead to sepsis that would be managed and survived by relaparotomy. Whereas, the cause of mortality in case 4 was due to delayed referral to the hospital and rapid worsening of the condition that lead to irreversible phase of sepsis that even relaparotomy could not save the patient’s life.

In conclusion, post cesarean PPU is a rare condition which may result in catastrophic maternal death. New onset tachycardia, abdominal pain and distension without congruent changes in hemoglobin should raise concerns for intra-abdominal emergency including PPU. A high index of suspicion by the obstetrician coupled with coordinated care by a general surgeon is necessary. Adjunct tools such as ultrasound and CT scan may contribute to a timely diagnosis and reduce maternal mortality rate.

## Data Availability

The datasets used during the current study are available from the corresponding author on reasonable request. They are divided in two group. The data of the patients that declared in the article and are available with more detail by corresponding author and can be sent by her. The second data group were extracted from public database like pubmed that were listed in the table with reference. The data are available to any scientist wishing to use them for non-commercial purposes, without breaching participant confidentiality.

## Abbreviations

PPU: Perforated peptic ulcer
NSAIDs: Non-steroidal anti-inflammatory drugs
ICU: Intensive care unit
Hb: Hemoglobin
NPO: Nothing by mouth
PP: Pneumoperitoneum
US: Ultrasound
PE: Pulmonary embolism
EKG: Electrocardiogram
